# An Overview of Indoor Localization System for Human Activity Recognition (HAR) in Healthcare

**DOI:** 10.3390/s22218119

**Published:** 2022-10-23

**Authors:** Luigi Bibbò, Riccardo Carotenuto, Francesco Della Corte

**Affiliations:** 1 Department of Information, Infrastructure and Sustainable Energy Engineering, Università Mediterranea di Reggio Calabria, 89060 Reggio Calabria, Italy; 2 Department of Electrical Engineering and Information Technologies, Università degli Studi di Napoli Federico II, 80125 Naples, Italy

**Keywords:** human activity recognition, indoor localization, human tracking, signal measurement, positioning algorithms, GPS, IPS

## Abstract

The number of older people needing healthcare is a growing global phenomenon. The assistance in long-term care comprises a complex of medical, nursing, rehabilitation, and social assistance services. The cost is substantial, but technology can help reduce spending by ensuring efficient health services and improving the quality of life. Advances in artificial intelligence, wireless communication systems, and nanotechnology allow the creation of intelligent home care systems avoiding hospitalization with evident cost containment. They are capable of ensuring functions of recognition of activities, monitoring of vital functions, and tracking. However, it is essential to also have information on location in order to be able to promptly intervene in case of unforeseen events or assist people in carrying out activities in order to avoid incorrect behavior. In addition, the automatic detection of physical activities performed by human subjects is identified as human activity recognition (HAR). This work presents an overview of the positioning system as part of an integrated HAR system. Lastly, this study contains each technology’s concepts, features, accuracy, advantages, and limitations. With this work, we want to highlight the relationship between HAR and the indoor positioning system (IPS), which is poorly documented in the literature.

## 1. Introduction

Human activity recognition (HAR) is the scientific field that studies the identification of movements or actions performed by a person through the detection of signals sent by wearable sensors or smartphones or through video frames or images. The activities are carried out indoors, such as walking, sitting, stairs, and standing. It is also essential to know where the practical activities are carried out. In interpreting human movement, computer technology and artificial vision are used [1]. HAR has multiple applications such as surveillance, anti-terrorist security, lifelogging, and assistance. These systems have proven very useful in providing efficient home care for the elderly and indoor tracking systems. The percentage of older adults currently continues to grow, consequently determining a need for assistance for those subjects who, losing autonomy and wanting to continue living in their own home, require continuous support in real time. Therefore, it becomes essential to note what the older adult does regarding daily activities. The basic principle is to monitor older people at home or in a nursing home. The system must be able to detect anomalies or deviations in daily activities that are indicative of a decline in people’s capabilities. It should also be able to detect emergencies [2]. A positioning system is necessary to ensure these systems’ effectiveness. The developments in artificial intelligence, wireless communication systems, and nanotechnologies have allowed the creation of intelligent home care systems, avoiding hospitalization, with an evident reduction in healthcare costs. The HAR system has three main components:

- The sensing module continuously collects information through sensors on the activities carried out.
- The processing and selection module extracts features that help discriminate between activities.
- The classification module uses the features to identify the individual’s activity.

The exact position of the subject can be associated with improving the system’s accuracy. Internal positioning systems are used to acquire this information.

The indoor positioning system (IPS) provides continuous real-time localization of objects or people within an enclosed space in different environments, using a network of transmitters and receivers. The value of these systems is identified in the following performances:

- Know in real-time how people move within a structure.
- Identify where a particular subject is.
- Activate alarms when particular situations are identified.
- Support security and emergency services to direct them where their intervention is needed.
- Track personnel at risk when they reach designated collection points in the event of an evacuation.

The technologies available today are different and allow a wide range of positioning accuracy from meters to millimeters. These techniques cover a wide range of applications: to ensure assistance to patients in need of assistance, for real-time home tracking of physical assets within industrial facilities or identification of assets in supermarkets, museums, or galleries, for the identification of a specific work of art, or, in the field of transportation and logistics, identification of the position of the objects to be shipped [3]. With the proliferation of the Internet of things (IoT) devices in the home, various IoT service scenarios have been proposed, including IPS [4]. In addition, IoT is a heterogeneous set of technologies and communication that makes it possible to connect any intelligent device, favoring human subjects’ connection for exchanging information.

Current positioning techniques are divided into systems for external and internal positioning. Regarding external positioning, with current techniques, good results in the accuracy of the position obtained have been achieved. For indoor positioning, the following issues must be taken into account in order to obtain precise results:

- The presence of obstacles weakens the signal (fast fading);
- The presence of obstacles creates the problem of signals not being in a direct line (not line of sight (NLOS));
- The structure and nature of the construction materials of the indoor environment may create the problem of reflection and refraction (multipathing), making it difficult to determine the correct origin of the signal;
- The climatic changes in the signal’s means of transport affect the propagation speed.

These conditions affect the propagation of electromagnetic waves; therefore, positioning techniques must consider this to improve indoor position performance.

For the outdoor environment, the most widely used system is the GPS (Global Positioning System) [5], a satellite-based global geolocation system.

In addition to the GPS, there are other satellite navigation systems such as GLONASS (Global Orbiting Navigation Satellite System) developed in the Soviet Union [6], BDS (Bei Dou Navigation Satellite System) developed in China [7], and Galileo, developed in Europe [8].

GPS satellites emit radio signals from which information is obtained relating to the distance between the satellite and a receiver on Earth and the time it takes for the signal to reach it.

From a structural point of view, the GPS consists of the following components:

- The space segment consists of a constellation of satellites.
- The control segment comprises ground stations with the task of synchronizing the clocks of all the satellites, knowing their position, and possibly correcting them.
- The user segment comprises an antenna capable of acquiring signals and a receiver capable of decoding and processing them.

However, GPS is an excellent system for obtaining the position of a receiver with good precision, and it can be used mainly in an external environment. Indoor, however, the accuracy drops dramatically due to the inability to penetrate most building materials. Currently, much of human life takes place inside buildings, and it is increasingly necessary to know the location of a mobile device. Inside buildings, due to the lack of line of sight, the loss of the signal, the multiple errors due to multipathing that are created, and the problem of inconsistent time delays, it is not possible to rely on the satellite system. This limitation hinders the implementation of GPS in indoor localization systems, although it can be solved using “high-sensitivity GPS receivers or GPS pseudoliths” [9]. Pseudoliths (contraction of “pseudo-satellites”) are RF transmitters that emit signals similar to GNSS, allowing a more accurate localization closer to the receivers than traditional GPS transmitters installed on board satellites. Transmissions reach places where GPS struggles typically, such as deep urban canyons, forests, and valleys. Pseudoliths can be placed where they can increase or replace GPS signals from satellites However, the cost of implementation can be an obstacle to this system’s application. Therefore, other technologies should obtain real-time information on humans and objects in indoor areas [10]. In the literature, there are different experiences on indoor localization [11]. There are different technologies capable of localizing objects or people indoors in an accurate manner, but this is achieved by using specialized and rather expensive hardware and sensors that must though be placed in advance within a confined space; such systems also reach sub-millimeter accuracies but to the detriment of usability, cost, and scalability.

Compared with outdoor localization, indoor localization is more complex because the indoor communication channel varies significantly with the environment and depends heavily on many factors, such as building structure, room layout, and construction materials.

Positioning systems can use 2D [12] or 3D [13] models. The latter has a higher positioning accuracy but requires higher costs due to the hardware infrastructure. The lower cost of solutions based on 2D models favors their wide use in applications where accuracy is not to be favored. The technologies usually used in 2D models are Bluetooth, ZigBee, and Wi-Fi. In 3D models, on the other hand, infrared, ultrawideband, and ultrasound are applied. In the field of solutions based on 2D models, the one on fingerprints [14] coupled with Wi-Fi systems applicable for outdoor and indoor positioning is widespread.

**Figure 1:**
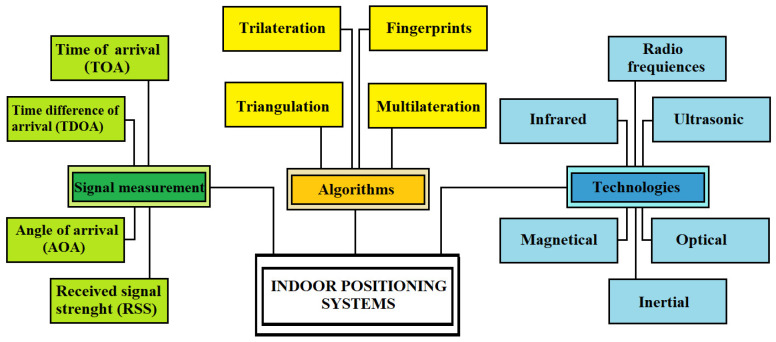
Indoor positioning systems.

There are different indoor positioning systems [15] depending on the technology used, and sometimes they can even be combined (Figure 1). There is no perfect solution; each has its strengths and weaknesses.

An efficient home care system must have characteristics that improve the quality of life; it is necessary to design a suitable environment to recognize where and what the elderly are doing successfully. The integration of the exact position of the person within the recognition system of human activities is essential to intervene promptly in case of unforeseen events and falls. Therefore, it is crucial to assist the elderly to avoid incorrect actions in case any behavioral changes are observed.

We have found that, in the literature, there is little experience on the effectiveness of the combination of IPS and HAR technologies. This work aims to explore possible systems that can offer a comprehensive solution. The methodology used in developing the work was based on the in-depth analysis of the activities’ multiple aspects of the localization and recognition approaches. We report the different technologies available and examine techniques and algorithms. We compared them with each other, highlighting advantages, disadvantages, and characteristic elements. We also research the testimonies on the integrated and compared systems to identify a possible solution that could satisfy different contexts. Lastly, the surveys confirm the issue’s complexity and the need for new research efforts. The work concludes with possible scenarios for future developments.

**Figure 2:**
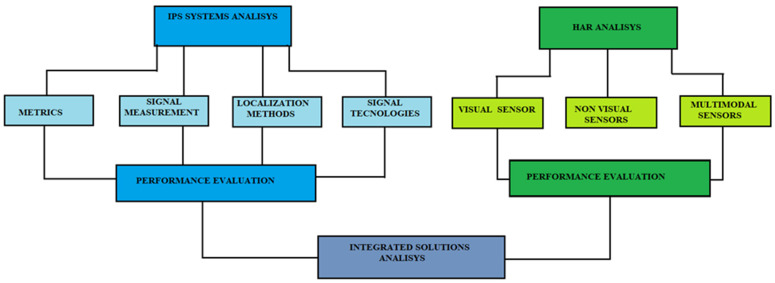
Diagram of relationships between the sections.

Section 2 describes the main features of an indoor positioning system; Section 3 analyzes the techniques used in localization; Section 4 illustrates the applied algorithms; Section 5 reviews the technologies; Section 6 describes the various applications developed; Section 7 concludes the paper. Figure 2 shows an outline of the relationships between the sections.

## 2. IPS

**Figure 3:**
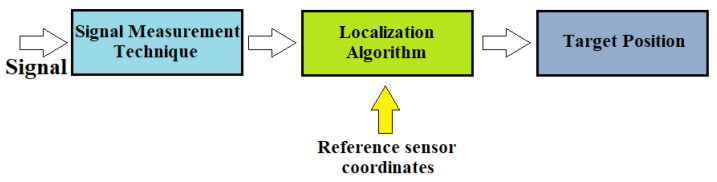
Diagram of the localization process.

The principle of operation of an indoor positioning system (IPS) uses reference sensor nodes with known positions that send a ranging signal to the mobile device attached to the target. Then, the mobile device perceives the request signals and issues a ranging reply to the reference sensor. At this point, the traveling time of the ranging signal between the transmitter sensor node and the moving target can be calculated. The measured data are transferred to a data center using positioning algorithms, and we can determine the target’s exact position. Therefore, for the localization of unknown nodes, we must first measure some metrics and then use the algorithm to calculate the position itself, as shown in Figure 3 [16].

In the first phase of the process, between fixed and mobile nodes, all the characteristic information of the signals is exchanged, such as direction, length, time of arrival, and coordinates of the referent node. In the second phase, however, the obtained parameters are transferred to the calculation algorithm to determine the exact position of the moving object.

Positioning systems can be distinguished according to the principle applied, such as proximity, range, and scene analysis.

**Proximity** uses the maximum received signal strength of an anchor node to calculate the location of the moving node. The near-field communication (NFC) technique is commonly used. We can easily implement the system; however, the accuracy is of a low level.

**The range** is based on measuring the distance of the communication signals. The location of the target node is determined either according to the direction or distance. For example, the angle of arrival (AoA) is usually applied for the first one. The time parameter (ToA or TDoA) or signal properties (RSSI) can be used for the second.

**Scene analysis** is a pattern recognition class of methods that uses the characteristics of a scene from a particular viewpoint to match patterns. The currently measured wireless characteristics are compared to prestored characteristics for each pattern to determine a match. We use the best match as the current location of the mobile node. The commonly used method is fingerprinting [14]. The system’s accuracy is excellent but burdensome, as it takes a long time to collect the characteristics needed for each pattern and requires substantial storage space. Moreover, changes to the environment may require the characteristics to be re-evaluated. In recent years, numerous indoor positioning technologies have been developed [17], which differ in the type of hardware used and the localization algorithm. Each has been developed for a specific need and has attributes that make it preferable in certain respects and characteristics or limited by its very nature if used for an application other than that assumed. To understand which of these technologies is the most suitable to apply in a specific context, it is necessary to create metrics against which to evaluate each technology.

### Evaluation Metrics

For the best choice of the most suitable system for the requested application, it is advisable to examine a series of performance indices [18]. For example, some applications may require low-cost IPS, while others may require high accuracy, such as healthcare systems. Below are the commonly used parameters.

1. **Accuracy** is the main feature that evaluates the average difference between the detected and actual positions (ground truth) [19]. Generally, this value is not fixed but oscillates concerning various parameters; thus, the reference is made to minimum and maximum values. Depending on the technology used, we can have the values in meters reported in Table 1.
2. **Coverage** indicates the functional surface within which the examined technology is effective. Depending on the type of technology used, it takes on different values. IPS coverage usually ranges from a few meters to scalable systems that can cover multiple large environments by adding hardware. In the case of challenging-to-scale technologies, this value represents the maximum local area covered, while, in the application of techniques that can scale (increasing their level of coverage), it represents the distance or area that can be covered by a single cell. Generally, technologies with more excellent coverage typically imply lower accuracy. In Table 1, the values of coverage are reported in meters.
3. **Scalability** indicates the possibility with which the technology can be extended, referring both to the coverage area and to the number of users supported simultaneously [20].
4. **Security and privacy** represent the level of control of access to the subjects’ personal information.
5. **Cost** includes all the costs necessary for the implementation and operation of the system, such as infrastructure costs, installation and maintenance, and energy consumption to run the components. The latter represents a fundamental parameter to ensure system continuity and higher mobility [21].
6. **Complexity** represents the level of complexity of designing, constructing, and maintaining an IPS.
7. **Support/infrastructure** represents the hardware necessary for the system to operate, i.e., if specific equipment is needed, or it can refer to the integration of an infrastructure located in the localization area, such as sensors or transmitters. The density and cost of these additional infrastructures weigh on the expansion capacity of the technology if it is necessary to use more nodes of the infrastructure.
8. **Continuity** indicates the property of continuous operation of an IPS over an appropriate time to perform its specific function, including acceptable outage frequency.
9. **Usability/user acceptance** represents how convenient and usable the technology is to the end user. A simpler infrastructure is easier to use.
10. **Privacy** is a crucial aspect to keep in mind that is not always carefully evaluated in IPS systems. Security mechanisms should be in place to improve user privacy, protecting data from intrusion or misuse [22].

From the analysis of the tabulated values, a broad scenario of adoptable solutions can be seen depending on the specific design needs. In particular, for accuracy, each technology has its own range of applicability, and none overlaps the other.

## 3. Signal Measurement Techniques

There are different approaches for this phase, one based on time, another on the receiving angle, and a third on the received signal strength [23].

### 3.1. Time-Based Methods

#### 3.1.1. Time of Arrival (TOA)

**Figure 4:**
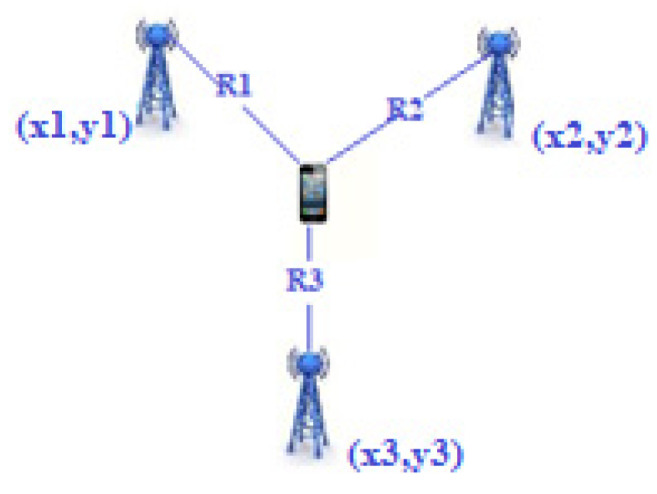
Time of arrival.

The time of arrival (TOA) [24] method calculates the distance between the emitter and the receiving node considering the time elapsed between the emission and reception of the signal. It is also called time of flight (ToF) because it measures the signal transmission time between the receiver and the transmitter. Knowing the speed of propagation of the signal in the middle and the time taken to get from one point to the other (time of flight or flight time), the space traveled is directly calculable as follows:

(1) $$R_{i}=v*t,$$

where *v* is the speed of the signal employed, which is the transmission time of the signal received by node *i*, and R*_i_* is the distance of signal transmission received by node *i*. Then, the location of the target is estimated using triangulation. For this method to be effective, it is necessary to have three reference points (also called anchors). Graphically representing the system, the node of which we want to calculate the coordinates would be given by the intersection of the three circumferences with the center of the reference nodes (Figure 4). In that case, we would have the following equations:

(2) $$\begin{array}{l} {\left(x_{1}-x\right)}^{2}+{\left(y_{1}-y\right)}^{2}=R_{1}^{2}\\ {\left(x_{2}-x\right)}^{2}+{\left(y_{2}-y\right)}^{2}=R_{2}^{2}\\ {\left(x_{3}-x\right)}^{2}+{\left(y_{3}-y\right)}^{2}=R_{3}^{2} \end{array},$$

where (*x*, *y*) are the coordinates of the target node.

This approach, however, has the big flaw of requiring the transmitter and receiver to be synchronized, which is not always feasible. To achieve high accuracy, this issue of the TOA technique can be compensated for by combining it with UWB (ultrawideband, a radio-based communication technology). This technology uses a short pulse duration to filter the signals caused by reflection to improve the overall performance.

#### 3.1.2. Time Difference of Arrival (TDOA)

With this technique, the arrival times of various reference signals are evaluated, and, from the difference in these times, it is possible to determine the location of the target node [25]. The difference in signal arrival time at two reference points is used to calculate the distance difference between the target and the reference points [26]:

(3) Δd=v∗(Δt),

where *v* is the speed of the signal employed, and Δ*t* is the difference in arrival time at each reference point. In two dimensions, this leads to the following form:

(4) $$\Delta d=\sqrt{{\left(x_{2}-x\right)}^{2}-{\left(y_{2}-y\right)}^{2}}-\sqrt{{\left(x_{1}-x\right)}^{2}-{\left(y_{1}-y\right)}^{2}},$$

where (*x*_1_, *y*_1_) and (*x*_2_, *y*_2_) are the known positions of the beacons.

**Figure 5:**
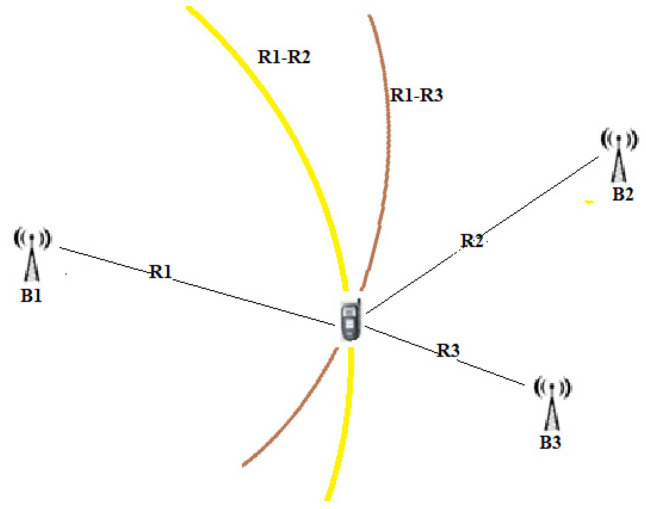
Time difference of arrival.

Unlike the TOA, the transmitted messages do not convey a timestamp because the difference in the time of receipt is already sufficient information to guarantee localization. Instead, through the multilateration technique, by comparing the arrival times of the signals in pairs, it is possible to build a system of hyperboles whose intersection determines our position (Figure 5). Unlike TOA, in the TDOA method, not all nodes need to be synchronized; it is sufficient that the anchor nodes are synchronized.

#### 3.1.3. Round Trip Time (RTT)

The RTT represents the time between sending a signal plus the time required to confirm that signal [27]. In some of its implementations, it is a technology that utilizes the fine timing measurement (FTM) protocol to measure time with picosecond resolution. It allows a mobile device to determine its distance from an access point (AP) by measuring the duration of a time interval of transmission of radio waves traveling back and forth between the transmitter and receiver, which are generally called the initiator and responder. With it, the distance of the target node (R) is obtained with the following equation:

(5) $$R=\frac{(t_{RT}-t)c}{2},$$

where *t_RT_* represents the time of the signal to travel from a node and vice versa, *t* represents the hardware’s default delay time, and *c* is the transmitted signal’s speed. This method solves the synchronization problem. Instead of using two local clocks in both nodes to calculate the delay, a single node is used to record the transmission and arrival times. Errors in estimating RTT distance and user location due to factors such as multipath fading occur most often in an indoor environment. The following factors that may be detrimental are related to the topology of the network infrastructure used for the connection between transmitter and receiver:

- Propagation delay, which depends on the distance between the transmitter and the receiver;
- Processing delay that depends on the number of nodes on the network. A node can also experience congestion by slowing the connection and increasing the RTT.

### 3.2. Receiving Angle

#### Angle of Arrival

**Figure 6:**
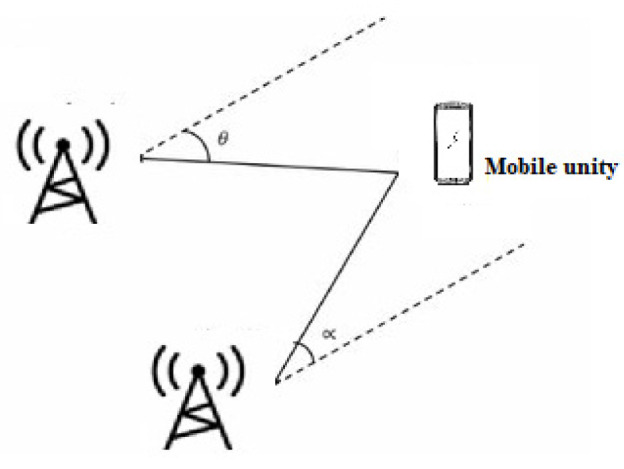
Angle of arrival.

This is based on determining the angles between the propagation direction of the received signal and two or more predetermined references [28]. At least two AOA measurements from two different references are necessary to estimate a mobile’s position location (Figure 6). To improve accuracy, three beacons or more are used for position estimation. This measurement can be made using directional antennas or antenna arrays. The position estimation is performed by comparing the carrier phase or signal amplitude across multiple antennas. With AOA, no time synchronization between nodes is required. This technique, however, has limitations because, as distance increases, accuracy decreases. It is affected by the phenomenon of multipath and NLOS.

### 3.3. Connectivity

#### Received Signal Strength (RSS)

**Figure 7:**
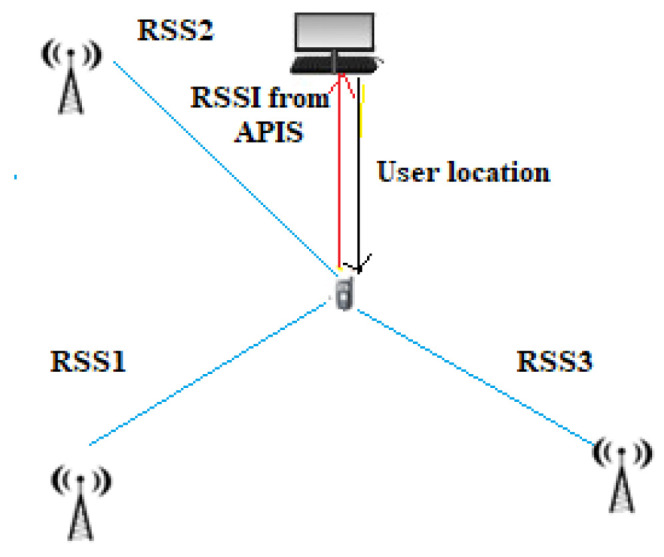
Received signal strength.

This method is based on the received power of a signal, measured in dBm, and on the relationship between the attenuation of the signal and the distance traveled (Figure 7). Knowing the power with which the signal is emitted and, of course, the power with which the signal arrives at the receiver, it is possible to calculate its attenuation. In fact, since the attenuation of the signal is directly proportional to the distance, with the use of theoretical and empirical models based on the law of signal propagation, it is possible to derive the distance once the attenuation is known [29]. Devices using this technique have the advantage of being low power consumption essential features in the case of WSN (wireless sensor network) nodes, which typically have limited power.

The Friis formula can be used to evaluate the signal strength received:

(6) $$P_{R}=P_{T}\frac{G_{T}G_{R}\lambda^{2}}{{\left(4\pi \right)}^{2}d^{n}},$$

where *P_R_* is the received signal power, *P_T_* is the transmitted signal power, *G_R_* is the gain of the receiving antenna, *G_T_* is the transmitting antenna gain, *λ* is the wavelength (*λ* = *c*/*f*, where *c* is the speed of wave propagation, and *f* is its frequency), *d* the distance in meters, and *n* the signal propagation constant, also called propagation exponent, dependent on the environment in which we are located.

Considering that the power of the transmitted signal decays with distance, we can modify the previous relationship with the log-normal shadowing model used in wireless communications [30]:

(7) $$P_{r}(d)=P_{0}-10n\log d/d_{0}+\zeta_{\sigma },$$

where *P_r_*(*d*) is the received power at distance *d*, *P*_0_ is the received power measured at reference distance *d*_0_, *n* is the path-loss exponent, and *ζ_σ_* is the zero-mean Gaussian noise.

Table 2 compares the different signal measurement techniques through their main characteristics.

## 4. Localization Methods

In the second phase, through the measured parameters of the signal and the known coordinates of the reference nodes, the coordinates of the unknown nodes can be determined. There are several positioning techniques; among the most important are trilateration, triangulation, multilateration, and fingerprinting.

### 4.1. Trilateration or True-Range Multilateration

**Figure 8:**
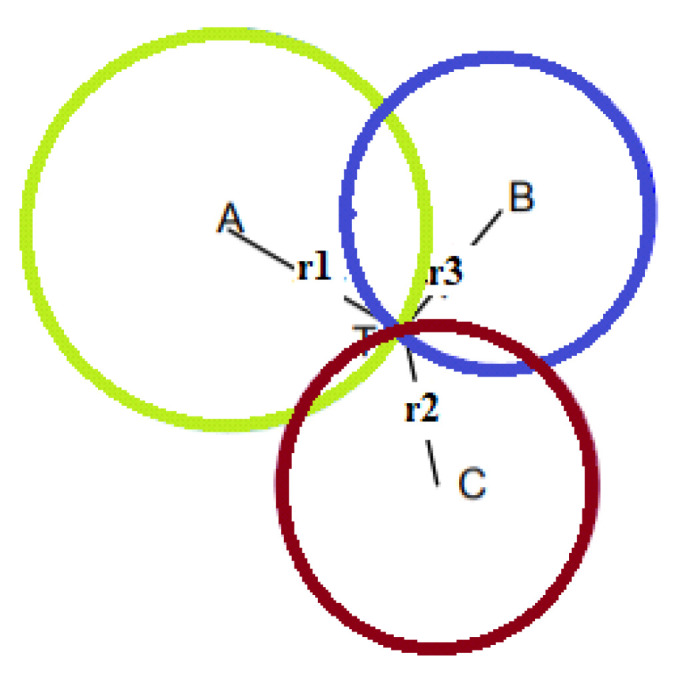
Trilateration.

Lateration, also called range measurement, computes the position of an object by measuring its distance from multiple reference positions. It is a technique that calculates the physical position of the target node by knowing the positions of the three fixed non-collinear referent nodes in the 2D space. Finding a location in two dimensions requires distance measurements from three non-collinear points. In three dimensions, distance measurements from four non-coplanar points are required. Using the geometry of the circles, it is possible to determine the positioning of the moving node [31]. Circles are drawn with the coordinates of the reference node as their center and radii equal to the estimated distance, for which, ideally, the target node is located at the point of intersection of the three circles (Figure 8). When more reference points are used than necessary, then we speak of multilateration or true-range multilateration. The circumference can be described through the following equation:

(8) $${\left(x-x_{i}\right)}^{2}+{\left(y-y_{i}\right)}^{2}=r_{i}^{2},$$

where (*x_i_*, *y_i_*) are land coordinates of the various centers, and *r_i_* is the radius of the *i*-th circle. Accordingly, the coordinates of the target node can be obtained by solving the following system:

(9) $$\begin{array}{l} {\left(x-x_{1}\right)}^{2}+{\left(y-y_{1}\right)}^{2}=r_{1}^{2}\\ {\left(x-x_{2}\right)}^{2}+{\left(y-y_{2}\right)}^{2}=r_{2}^{2}\\ {\left(x-x_{3}\right)}^{2}+{\left(y-y_{3}\right)}^{2}=r_{3}^{2} \end{array}.$$

In practice, since the distance estimated is never perfect, the coordinates of the target node are rarely found at the point of intersection of the three circles; hence, it is likely that it is located within the area of intersection between the circles or even that the circles do not touch. To overcome this drawback, the AML (adapted multilateration) method developed by Kuruoglu et al. can be adopted [32] based on an iterative process applied to the three beacon nodes. Two circles are drawn around two randomly chosen beacon nodes. If the intersection point is unique, the coordinates of this point are acquired. If the circles do not touch, the spokes are increased proportionally to touch each other in one place. If, on the other hand, the two circles touch each other at two points, the one that has a distance from the third beacon closest to the estimate of the distance calculated for this node is chosen. At the end of the iterative process, the average of the coordinates that will determine the position of the target node is calculated.

### 4.2. Triangulation

**Figure 9:**
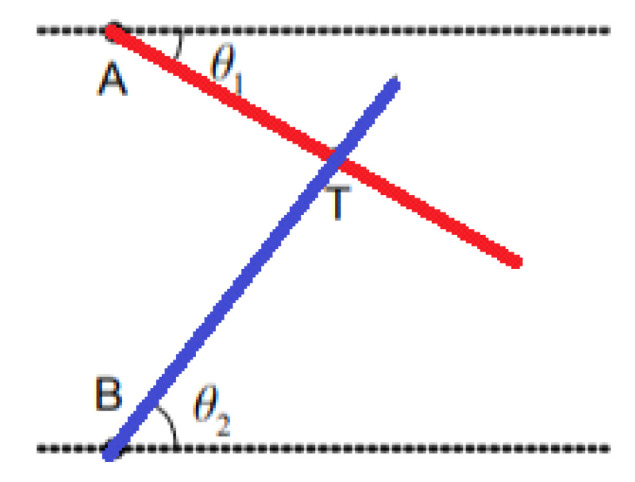
Triangulation.

Triangulation uses the geometric properties of triangles to compute object locations [33]. When the AOA measurement is available, the triangulation technique can be used to estimate the location of the target node using trigonometric laws [34]. The node at unknown coordinates estimates its angle to each of the two reference nodes and, on the basis of these angles and the positions of the reference nodes, computes its position using simple trigonometrical relationships (Figure 9). The position of the target node can be determined by the intersection of the direction lines of the pair of angles formed concerning the reference nodes (A, B). After obtaining the angles θ_1_, and θ_2_, the physical position of T, which represents the target to be located, can then be calculated on the basis of the predetermined coordinates of the reference nodes. In three-dimensional angulation, obtaining a precise position requires one length, one azimuth, and two angle measurements. Triangulation is a complex technique, requiring knowledge not only of the position of beacons but also of their spatial rotation. Therefore, calculations are not more complex than trilateration.

### 4.3. Pseudo-Range Multilateration

Pseudo-range multilateration, also known as hyperbolic positioning, is the process of locating an object by accurately calculating the difference in arrival time (TDOA) of a signal emitted by the object to three or more receivers [35]. In this case, the time of emission of the traveling signal is unknown. This technique bases the estimation of the position of a node on the minimization of the difference between the estimated distance via TDOA and the actual distance obtained from the known coordinates. Considering that the time of arrival is proportional to the space traveled by the signal itself, it is possible to identify the position of a target. For example, consider a target that emits a signal in an unknown position with *x*, *y*, and *z* coordinates in an area equipped with a multilateration system with *n* receivers (*P*_1_, *P*_2_, …, *P_n_*). The time the signal takes to reach each receiver by the emitter is given by dividing the space by the signal speed assumed as the speed of light *c*.

(10) $$\begin{array}{l} T_{1}=\frac{1}{c}(\sqrt{{\left(x-x_{1}\right)}^{2}+{\left(y-y_{1}\right)}^{2}+{\left(z-z_{1}\right)}^{2}})\\ T_{2}=\frac{1}{c}(\sqrt{{\left(x-x_{2}\right)}^{2}+{\left(y-y_{2}\right)}^{2}+{\left(z-z_{2}\right)}^{2}})\\ T_{3}=\frac{1}{c}(\sqrt{{\left(x-x_{3}\right)}^{2}+{\left(y-y_{3}\right)}^{2}+{\left(z-z_{3}\right)}^{2}})\\ ...........................................\\ T_{n}=\frac{1}{c}(\sqrt{{\left(x-x_{n}\right)}^{2}+{\left(y-y_{n}\right)}^{2}+{\left(z-z_{n}\right)}^{2}}) \end{array}.$$

Assuming that the point *P_n_* in 3D coincides with the origin of the system, we have that *T_n_* is expressed as

(11) $$T_{n}=\frac{1}{c}(\sqrt{x^{2}+y^{2}+z^{2})}.$$

The differences in the time of arrival concerning the reference site are

(12) $$\begin{array}{l} \tau_{1}=T_{1}-T_{n}=\frac{1}{c}(\sqrt{{\left(x-x_{1}\right)}^{2}+{\left(y-y_{1}\right)}^{2}+{\left(z-z_{1}\right)}^{2}}-\sqrt{x^{2}+y^{2}+z^{2}})\\ \tau_{2}=T_{2}-T_{n}=\frac{1}{c}(\sqrt{{\left(x-x_{2}\right)}^{2}+{\left(y-y_{2}\right)}^{2}+{\left(z-z_{2}\right)}^{2}}-\sqrt{x^{2}+y^{2}+z^{2}})\\ \tau_{3}=T_{3}-T_{n}=\frac{1}{c}(\sqrt{{\left(x-x_{3}\right)}^{2}+{\left(y-y_{3}\right)}^{2}+{\left(z-z_{3}\right)}^{2}}-\sqrt{x^{2}+y^{2}+z^{2}})\\ ......................................................................\\ \tau_{n-1}=T_{n-1}-T_{n}=\frac{1}{c}(\sqrt{{\left(x-x_{n-1}\right)}^{2}+{\left(y-y_{n-1}\right)}^{2}+{\left(z-z_{n-1}\right)}^{2}}-\sqrt{x^{2}+y^{2}+z^{2}}) \end{array},$$

where (*x_i_*, *y_i_*, *z_i_*) with variables from 1 to *n* are the known locations of the various receivers. Each of the above equations represents a hyperbola; from the system’s resolution, the emitter coordinates are obtained.

**Figure 10:**
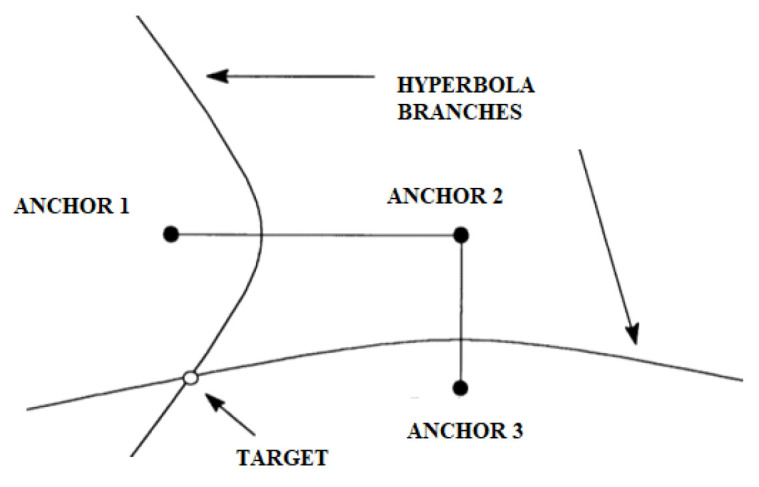
Intersection of hyperboles that identify the position of the target.

From a graphical point of view, the coordinates of the source can be determined by the intersections of the above hyperbolas, as shown in Figure 10. A hyperbola is defined as a geometric place of the plane having as a constant the difference in distances from the foci that, in our case, are the reference sensor that is the first to receive the signal from the target and the umpteenth sensor that receives the same signal.

In addition, to avoid conflict between the emitters, a minimum separation distance between the various nodes must be ensured by defining a protection zone around each node so that any other node does not infiltrate it.

### 4.4. Fingerprinting

**Figure 11:**
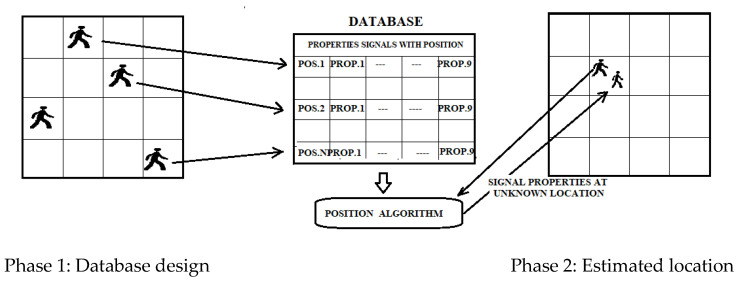
Diagram of the fingerprinting process flow.

Fingerprinting is a popular method of localization because of its good accuracy compared to other methods [36]. Traditional fingerprinting is based on RSSI but can also be based on static magnetic field measurements. This technique consists of two phases: a first offline phase of scene analysis and sampling, and a second online phase of pattern matching. In the offline phase, the signals from the different access points are acquired and subsequently stored in a database with the coordinates of the client device. This information can be deterministic or probabilistic. For example, in the online tracking phase, the user’s estimated position is obtained by matching the positions stored in the fingerprint database (Figure 11). Such systems may provide an average accuracy of 0.6 m [37]. Any change in the environment, such as removing or adding furniture, changes the “fingerprint” corresponding to each location; hence, the fingerprint database needs to be updated.

## 5. Signal Technologies

The communication platform used in WSN systems is crucial in assessing that the accuracy of elderly care systems is between 0.5 m and 1 m. The value of the update rate must be at least 5 s. The classification can be made according to the main medium used to determine the position. The technologies used are radiofrequency signals, ultrasound, infrared, optical signals, and inertial measurements.

### 5.1. Radiofrequency-Based Systems (RF)

**Figure 12:**
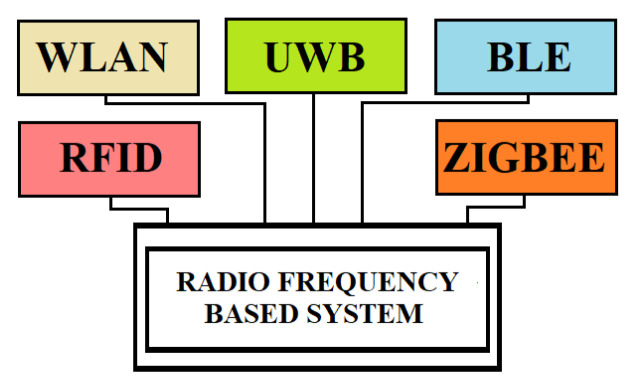
Radiofrequency-based systems.

Radiofrequency communication is wireless communication with electromagnetic wave frequencies ranging from 3 kHz to 300 GHz [38]. Frequency affects its capabilities such as coverage, penetration, and resistance to obstacles (Figure 12). Therefore, there are three categories of wireless technologies used for different applications: long-distance wireless technology, medium-distance technology, and short-distance technology [39]. RF-based positioning systems can cover long distances because they use electromagnetic waves that are not disturbed by the presence of objects or people. On the basis of this technology, networks such as RFID (radiofrequency identification), WLAN (wireless local area network), Bluetooth, and UWB (ultrawideband) have been created. The first few can be classified as narrowband-based technologies, while the last is a wideband-based technology.

RFID-based systems use two basic components: readers and tags. Tags can be active or passive depending on the power source. For example, the power supply of a passive tags comes from the electromagnetic energy transmitted by the nearest RFID reader. It cannot send data but only receive it. Active tags are powered by their battery and have a range of action up to 100 m from the reader [40]. Therefore, active RFID tags are helpful for long-range localization and object tracking. However, active RFID technology is unreliable for submeter accuracy and unavailable on many mobile devices. Passive RFID tags do not integrate the battery and backscatter received signal from the base station. Moreover, they are low-cost and require only a chip tag and an antenna. They are suitable for submeter detection and have a detection range of up to 10 m [41].

**WLAN** often operates according to the standard IEEE 802.11 at 2.4 GHz within the ISM band and has a range of about 50–100 m; however, the 5 GHz is now widely used for transmission due to less interference, less noise, higher constant connection, and higher speed [42].

The above IEEE standard is also known as Wi-Fi from the trademark name of the Wi-Fi Alliance. Wireless local area network (WLAN) technology allows you to connect to a network using radio waves. From an architectural point of view, it can be compared to a local small-scale cellular coverage network with radio transceiver devices such as access points (APs). To increase the connectivity range of a single access point (approximately 100 m) and, thus, be able to cover a larger area, more access points are commonly used, connected with information exchange entirely via radio interfaces, but with a loss in the system’s spectral efficiency. Its wide diffusion is due to mobile devices such as laptops, tablets, mobile phones, and other devices capable of connecting to a wireless network. Wi-Fi technology has an advantage over other technologies thanks to the presence in multiple environments of Wi-Fi access points, and the widespread use of mobile devices enabled by this technology [43]. Therefore, its advantages are as follows: widely distributed hotspots that make indoor positioning services widely usable; wide access freedom, which, thanks to the widespread distribution of existing Wi-Fi infrastructure, does not require network expansion with an apparent reduction in costs; the signals are not severely affected by not line of sight (NLOS). Most WLAN positioning systems are based on RSS, combined with the fingerprinting technique. In general, Wi-Fi positioning techniques can be categorized into four groups: RSSI based, fingerprint-based, AOA-based, and TOF-based. Positioning based on fingerprinting and RSS is the most accurate method but involves building a database, resulting in a more significant workload, and it requires about three or four Apps per 100 m^2^, making it more expensive. Continuous Wi-Fi scanning consumes a substantial amount of battery power, rendering it disadvantageous for long-term use. This technology is very flexible since, within its radio coverage, nodes can communicate without restrictions. Radio waves can penetrate walls, and senders and receivers can be placed anywhere. Wi-Fi enables the addition of additional users.

**BLE** (Bluetooth Low Energy) is one of the low-power connectivity standards that operates in the 2.4 GHz ISM band. It can connect devices over a relatively short range, 70–100 m, with 24 Mbps. BLE beacons have the following characteristics: small size, cost-effectiveness, use their battery as a power supply, and can approximately calculate the distance to the beacon, thus estimating the user’s internal location if in the range of more than two beacons [44]. It does not need expensive hardware for accurate localization, and direct LOS is unnecessary. Theoretically, up to 10 cm accuracy can be achieved at distances between beacons and anchors less than 1 m in low-noise environments. BLE is designed with very short ranged wireless transmissions. Adjusting the transmission power makes it possible to extend the range up to 100 m. Although both Bluetooth and Wi-Fi operate in the 2.4 GHz frequency band, there is generally no interference between the signals. Therefore, both technologies can be used in positioning systems to obtain better results by combining their best performances [45]. RSSI and trilateration algorithms are used to determine the exact position of the mobile device [46]. It is a competitive technology thanks to its low energy consumption and good level of accuracy. The latter depends on the Bluetooth node’s stability and indoor propagation’s environmental conditions.

**UWB** is a radio technology for short-range communications with high bandwidth greater than 500 MHz and a carrier frequency greater than 2.5 GHz, featuring low power consumption [47]. Its large bandwidth allows obtaining a high data transmission speed, short wavelength, and high temporal resolution. Another valuable property of UWB is that signals can easily pass through obstacles. These features have made UWB suitable for indoor wireless positioning. The detection of TOA and time difference of arrival (TDOA), which allow a higher accuracy than other localization algorithms due to the high temporal resolution of UWB signals, where the multipath effect is minimized, are applied to calculate the distance between a reference point and the target. In addition, it is possible to add UWB beacons to existing Wi-Fi infrastructure. The hybrid method combines the availability of Wi-Fi infrastructure, which reduces cost, and the accuracy of UWB when deploying their algorithm; in such hybrid systems, the localization error is limited to 20 cm [48].

**ZigBee** is a specification based on the IEEE 802.15.4 standard [49]. It uses the 868 MHz bands in Europe, the 915 MHz bands in the United States and Australia, and 2.4 GHz in other regions. ZigBee technology is mainly used for applications belonging to integrated systems provide low transmission speed and low consumption. The ZigBee protocol allows the network to have a reduced expenditure of energy resources by exploiting only the energy contained in the battery incorporated in the individual nodes.

The characteristics of this protocol are IEEE 802.15.4 standard, a speed of 250 kbps, coverage of 10–100 m, and type mesh. It is a short-range communication standard such as Bluetooth and Wi-Fi, which covers a range of 10 to 100 m but differs from those used for high-speed data transmission communication.

**Figure 13:**
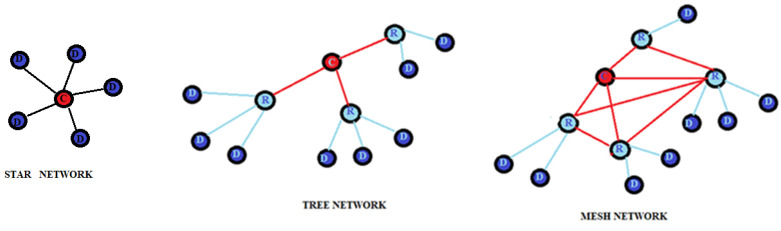
Network topologies.

The ZigBee IEEE 802.15.4 standard defines two distinct types of devices: FFD (full function device) nodes that can perform all the functions defined by the ZigBee standard and RFD (reduced function device) nodes that can perform only a limited number of functions; in particular, they are nodes that cannot forward traffic to the other nodes, but only act as sources or final recipients of traffic. Communication equipment of the ZigBee protocol is divided into three types: coordination equipment, routers, and terminals. The coordinator is an FFD and is responsible for the overall management of the network, starting the network, setting the parameters of the network, and transferring the packets of the application. It can also be used as a router. The router is used in tree and mesh topologies to expand network coverage. It is usually located either in the central area of the network. Its function is to find the best route to the destination to transfer a message. It can perform the same functions as the coordinator except for the start of the network. The terminal device can be an RFD responsible for collecting and transmitting data. ZigBee nodes consume almost no energy when at rest. ZigBee technology provides three structures: star, tree, and mesh. The star structure is the simplest and consists of a coordinator and a few end devices. The end devices are all connected to a single coordinator node, and all communications pass through this coordinator. The tree topology consists of a coordinator, a few routers, and end devices. Routers allow extending network coverage; end devices are connected in groups to each router and can only communicate with their coordinator. In the case of disabling the latter, the nodes connected to it remain isolated and cannot communicate even with the nearest nodes. The mesh topology, a peer-to-peer network, consists of a coordinator, several routers, and end devices. A mesh topology is self-healing, which means that the node will find an alternative path to the destination during transmission if a path fails. The range of the network can be easily changed by adding or removing multiple devices. Figure 13 shows the three topologies. Commonly, the RSS signal is used to estimate the distance between two or more ZigBee sensor devices [50].

### 5.2. Ultrasound-Based Systems

Ultrasonic systems are most commonly used in short-range measurement [51]. The signals have several advantages, such as a slow propagation speed, a negligible penetration in walls, and a low cost of the transducers. They do not interfere with electromagnetic waves and have a relatively short range. Instead, they use building material and air as a propagation medium. The accuracy achieved by ultrasound-based systems is typically a few centimeters. These characteristics are attractive for use in indoor positioning systems. The distance between the beacon and the target node can be calculated by measuring the time of flight (TOF) of the ultrasonic signal, while the Friis formula can be used to evaluate the power budget and the SNR at the target. Using ultrasonic signals, the measurement of time is more accessible than in the case of radio waves due to the lower speed of the acoustic waves and, therefore, the lower temporal resolution required for the measurement itself. The TOF measurement requires a correct temporal synchronization of the network nodes [52].

The target coordinates can be estimated by multilateration concerning some beacons distributed in known places. These systems are called acoustic measurement systems because the systems function using sound waves. Ultrasound is stealthy for the human ear compared to the sound wave. Additionally, this system is associated with RF technology to fulfill the synchronization requirement [53]. Ultrasound has, in addition, several advantages over electromagnetic waves when the application of implantable devices in healthcare is considered. The presence of water in the human body produces attenuation of the RF waves; therefore, it is necessary to increase the transmission power. This increase can be translated into soft-tissue heating because of absorption. This factor increases the size and weight of an implantable sensor. On the other side, the choice of ultrasound technology allows for the creation of implantable sensors at the level of micro-dimensions and low energy consumption [54] due to the relatively low attention to transmitting power to implanted sensors encountered in the human tissues. Furthermore, they are not affected by interference, in the transmission of data for patient monitoring, due to the presence of other electromagnetic devices.

### 5.3. Infrared-Based Systems (IR)

Infrared (IR) radiation is electromagnetic radiation, the wavelength between 700 nm and 1 mm is greater than that of the visible spectrum but less than that of radio waves [55]. Its main advantage is its wide availability since many devices are equipped with IR sources. However, as they require line-of-sight communication and fail to penetrate opaque obstacles such as walls, their use is limited in individual rooms. It is a very reliable system as light cannot pass through the walls; hence, it is impossible for a tag to detect light from an anchor without being in the same room. It is, however, subject to interference from other sources of IR devices or possibly blinded by direct sunlight. For precise localization, many anchors are required, and we can have difficulties due to the low quality of the signal strength measurements required to calculate the position. Mobile node localization can be used as a measurement method for estimating the angle of arrival of the IR signals [56]. These systems can be divided into two types: direct infrared systems and diffuse infrared systems. The former uses a point-to-point data transmission standard achieving low-power communication. It requires line of sight (LOS) communication or a very short distance between the devices. The latter has a stronger signal than direct IR; it has a more extended reach (9–12 m). It uses wide-angle LEDs, which emit signals in many directions [57]. Another system tested is based on a single photodiode (PD) and multiple LEDs; it uses the angular diversity transmitter, which consists of multiple LEDs and a biconvex lens [58]. Furthermore, systems based on mixed IR and ultrasound [59] or acoustic signatures have been proposed [60].

### 5.4. Magnetic Field-Based Systems

This technology is used for low-frequency localization. It is based on a platform with a reference station that radiates a magnetic field and a magnetic sensor capable of receiving the radiated field. It is a system that measures position using the Earth’s magnetic field disturbances caused by steel structural elements in a building. Their presence deforms the geomagnetic field in a way that varies spatially but is temporally stable. In known positions, land variations of the geomagnetic field in the indoor environment are recorded to build magnetic maps to be used as fingerprints to identify the positions of an unknown target [61]. It is a method that combines the magnetic field measured by the sensor when the user is in a specific position with the fingerprints present in the magnetic map to estimate the user’s position. However, the accuracy of the location of the magnetic fingerprint can be influenced by the smartphone’s orientation and affected by geomagnetic storms. In addition, it depends on the accuracy of the localization of the magnetic field, the density of fingerprints, and the quality of the acquisition and maintenance process of magnetic maps. Therefore, the current magnetic field positioning technology is mainly combined with other indoor positioning technologies such as Wi-Fi [62] or inertial sensors (IMU) [63] to improve the accuracy. A significant advantage of this type of system is that it offers high accuracy and is not affected by most obstacles; hence, multipath or non-line-of-sight (LOS) errors are avoided. To these must be added the advantages of achieving safety, reliability, and a low cost without additional infrastructure requirements.

The system can use the environmental magnetic fields produced by iron inside reinforced concrete structures of modern buildings that create local variation in Earth’s magnetic field (geomagnetism). Generally, a nonuniform magnetic field produces different magnetic observations depending on the path used. Therefore, positioning can be determined using fluctuations in these environmental magnetic fields. An optimized compass chip inside a smartphone can sense and record these magnetic variations to map indoor locations [64]. Magnetic fields can be generated by a coil powered by alternating current (AC) or pulses of direct current (DC). Electromagnetic fields can also be used to obtain a position by combining the use of electric fields and magnetic fields. The two sources of fields are static charges that produce electric fields and currents which produce magnetic fields. Oscillating charges produce both magnetic and electric fields.

### 5.5. Optical System

Optical positioning systems can be divided into two main categories: systems in which a moving sensor must be located, whereby reference information is required, and systems in which fixed cameras detect images of moving objects without reference information. In the first case, it is possible to use LEDs that emit light as markers and a series of sensors photodiodes mounted in predefined positions that measure the angle of arrival of the light so that each segment of the space of interest falls within the field of vision of two units [65]. The data collected by the fixed sensors allow for calculating the position of the marker. In the second case, the images acquired by the cameras are combined with computer vision technologies to obtain the object’s positioning [66]. Multiple static cameras can track objects with a high update rate. The obtained accuracies are of the order of tens of micrometers, and the cost of the system increases.

### 5.6. Inertial System

**Figure 14:**
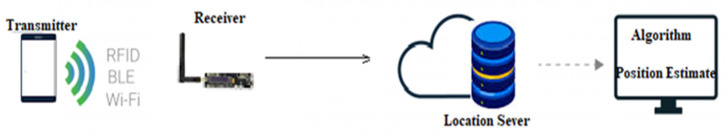
Architecture of IPS System.

An inertial system represents the positioning solution based on an inertial measurement unit (IMU) sensor. IMUs combine accelerometers (for measuring linear acceleration), gyroscopes (for measuring rotational speed), and optionally, a magnetometer (for measuring magnetic direction). This information can accurately determine orientation, velocity, and position [67]. This technique qualifies for accuracy, energy, and efficiency, provided that the inertial sensor is attached to the subject’s body. However, the measurements are prone to errors and require using Kalman-type filters. In addition, its cost is relatively high and often requires implementing the network infrastructure. By processing signals from these devices, it is possible to track the position and orientation of a device. The double integration of measured acceleration provides the relative position of an object concerning an initial position. Absolute positioning information may be obtained by fusing INS with complementary sensors. The signal-less localization method is based on mobile sensors such as accelerometer, gyroscope, magnetometer, and barometer, to track users by continuously estimating their displacement using dead reckoning [68]. With this technique, a sensor node uses its previous calculated position for localization at successive intervals. Although dead reckoning can provide the best available information about the current location with simple algorithms, it is subject to significant approximation errors. Another hybrid solution is to host the fake Wi-Fi printing with inertial sensors, overcoming the drawbacks of standalone solutions [69]. Figure 14 shows an architectural schema of an IPS application.

Table 3 compares related indoor localization systems.

## 6. Systems for HAR

Human activity recognition (HAR) is an application area aimed at determining people’s activities and the context in which they are addressed. In HAR systems, different technologies based on visual, nonvisual, and multimodal sensors are used to analyze and process data and images necessary to understand people’s behavior. Some analyses of the activities performed are carried out by human operators with the help of camera networks. This solution is currently used in security and surveillance services to visualize people’s behavior and detect anomalies to activate intervention services [70]. With the progress made by the new generations of cameras and intelligent classification systems, the contribution of operators is being reduced or even replaced with automatic systems, improving the efficiency and effectiveness of observation and the analysis process. The availability of these systems has found wide application in the home care field, making up for the shortage and expensive availability of caregivers. The use of visual sensors in the field of the healthcare system has been very effective in fall detection, especially for the elderly who suffer from cognitive loss. The results based on this technology stand at accuracy values of 99%.

Nonvisual systems are predominantly used in ambient assisted living (AAL) applications. Sensors are used that can collect a lot of information. They can be environmental detectors, microphones, or wearable devices. The collected data are sent to intelligent systems for classification. The solution based on multimodal sensors uses different sensory devices distributed in different parts of the body to collect real-time data to be analyzed for monitoring the health conditions of the elderly. The availability of smartphones equipped with multiple sensors: inertial, environmental, heart rate, and respiration rate has made these devices a suitable platform for collecting real-time data for innovative HAR solutions. These sensors offer a guarantee of privacy, are more efficient, and provide accurate physiological data but are not easily accepted by the elderly for wearability problems. Their misuse can lead to erroneous assessments and trigger signs of false emergencies. Table 4 shows a comparison between the three different types.

HAR systems represent the most suitable technology to monitor and assist the elderly and disabled people in ensuring their safety and wellbeing. They aim to identify the activities the elderly carries out daily regarding domestic work, personal hygiene, and free time. These activities implicitly identify where the older person is, as they are closely related to specific rooms within the house. Continuous real-time monitoring of activities and physiological data allows the identification of potentially dangerous situations. The precise identification of the patient’s whereabouts facilitates the interventions of health assistants in case of emergency or urgency. Therefore, by combining an IPS system, we have additional information to estimate a more accurate positioning and improve the recognition of activities. Combining these two themes requires an intelligent environment to deduce where and what a person is doing successfully. In addition, infrastructure-based solutions use dedicated equipment installed in buildings, which increases implementation costs but produces more accurate estimates; solutions without infrastructure reduce localization accuracy with lower costs.

HAR, developed to assist the elderly, can be seen according to different approaches such as health monitoring, ambient assisted living (AAL) for smart homes, and security and surveillance applications. Healthcare monitoring systems are designed to monitor the situations of individuals and provide valuable tools for emergencies. For this purpose, there are several functions, such as human tracking, fall detection, security alarm, and cognitive assistance. Wearable sensors and environmental sensors are used.

**Figure 15:**
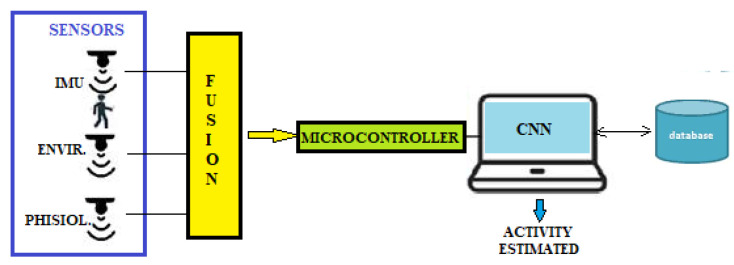
HAR system.

Ambient assisted living (AAL) involves using AR techniques to ensure that older people in the home remain safe and lead independent lives. In a smart home, devices are user-centric and integrated into the living environment of individuals. Cameras and inertial sensors generally monitor their activities and interaction with the environment. For the elderly, it is an advantage as it helps prevent, treat, and improve their wellbeing. Figure 15 shows the architecture of a HAR system.

The evolution of artificial intelligence technology has had a substantial impact on the development of forecasting models. This factor has favored using deep learning technology in constructing forecasting models within HAR systems. Their approach differs from statistical models, even though the two systems have the same aims. Both are used for forecasting and data mining. The statistical model is a mathematical function used to approximate reality. It is used to construct a data representation and to analyze any existing relationships between variables by establishing scale and significance. It is, in fact, based on the acquisition of raw data and its transformation into information that can be used to create a basis of a statistic learning model.

On the other hand, machine learning is based on the ability to learn from data instead of using preprogrammed algorithms, as well as use data to build and improve forecast models and evaluate performance based on new data that has not yet been learned. Statistical models to explain the relationships between variables must use hypotheses, confidence intervals, and inferences to validate hypotheses. Statistical data operate on smaller volumes of data than those used by ML models. The accuracy of statistical models is lower than that of ML models due to the impossibility of creating complex relationships between data. Being complementary, the tendency is to make the two systems coexist.

In machine learning, the federated learning model represents an innovative approach within HAR systems [71]. It represents a machine learning technique that allows the formation of predictive models by training an algorithm on multiple decentralized edge devices or servers that contain local data samples without exchanging them. Furthermore, it has the peculiarity of using decentralized private data without centralized collection. Federated learning is a machine learning technique in which the model is created without data sharing between users. The model can be downloaded by each user with their mobile device and updated with their local data. Local updates are then aggregated into the global model through an iterative process. This technology helps overcome all issues associated with privacy, data security, and access authorization. A peculiarity of this technology is the possibility of using different signal transmissions for the same type of activity. The need for frequent communication between nodes during the process requires sufficient computing power and memory as well as high-bandwidth connections to exchange model parameters.

To improve localization prediction subject to uncertainties due to the variables at play, Jamil et al. [72] implemented an accurate compensation mechanism on a combined PDR and BLE positioning system. The hidden Markov model was used for recognition of activities. The approach by correcting the involuntary acceleration of the body and the distortion of the magnetic sensor accurately determines the speed and position.

Taking into account the characteristics of positioning systems and the performance needs of a HAR system, it follows that integrated monitoring, localization, and tracking system should have the following characteristics: low power consumption, high accuracy, sufficient coverage, easy scalability, short return time, less computation, low cost, noncomplex infrastructure, and insensitivity to obstacles.

The solution must have the right compromise about the design needs; there is currently no technology that meets all the characteristics mentioned.

### 6.1. Intelligent Metasurfaces

In this subsection, an attractive, innovative technology is reported: intelligent metasurfaces, representing an emerging research topic that involves various disciplines, with digitization, programmability, and intelligence capabilities. Intelligence refers to the extraordinary property of making decisions, self-programming, and performing tasks without human intervention. It is an attractive technology for its potential to configure the propagation environment, making it more controlled.

In wireless transmissions, a penalizing phenomenon for radio waves is represented by the presence of the adverse effects produced by the propagation of incident EM waves; walls, ceilings, floors, and objects act as scatters, creating multipath paths. Controlling the propagation of radio waves can mitigate these adverse effects. Research in recent years in wireless technology has led to the creation of reconfigurable intelligent surfaces (RISs). These metasurfaces can counteract the adverse effects caused by path loss, signal absorption, and multipath fading, effectively controlling the EM response in reflected, refracted, and diffracted waves.

A metasurface is an artificial planar structure that contains repeated conductive elements, i.e., meta-atoms, on a dielectric substrate. The meta-atom is integrated with tunable functional materials and is designed to be reprogrammable. There are two different topologies of these surfaces: a programmable thin wallpaper and a programmable thin glass. Both have the following characteristics:

- Do not emit new radio waves;
- No power amplification;
- Low power consumption for operation;
- Low processing capacity for surface configuration.

It is a technology that is applied in different sectors. Metasurfaces can be installed in buildings to improve coverage, increase spectral efficiency and reduce exposure to EM radiation [73]. They can also be used on cars, trains, and planes to improve communication between infrastructure and carriers while reducing passenger exposure to the EM field. In smart homes, RISs can be used to improve device connectivity. In smart clothing, the meta-atom is integrated with tunable functional materials and is designed for reprogrammable clothing. RISs are used to make wearable body networks for monitoring people’s health. In the field of communications, this enables the creation of wireless networks that can be reconfigurable to adapt to changes in the surrounding environment.

In wireless transmissions, RISs can be used as antennas to replace conventional antenna arrays because they do not need to process incoming signals to send them to the intended receiver. Instead, they reflect the signal transmitted directly from the base station to the receiver. They are dynamic but passive elements that function as reflectors and can be controlled individually, whose phase response can be adjusted and optimized for beam orientation and focus. This solution is advantageous as it allows efficient communication, reducing the power consumption and hardware cost of conventional phased arrays [74].

Li et al. [75] studied intelligent metasurfaces tracing their future development as an intelligent platform for data mining, communication, energy harvesting, and sensing by directly processing illuminated information-carrying waves.

They explored the applications of intelligent metasurfaces in developing new wireless architectures and intelligent sensing.

Artificial intelligence is the core of intelligent surfaces, and the combination with deep learning techniques has made it possible to design intelligent devices and systems. Cui et al. [76] newly proposed coding and programmable metamaterials, where each meta-atom has a finite number of quantized physical states and can be used to encode digital information. The intelligent metasurface integrates the reprogrammable coding metasurface with deep learning algorithms by acquiring the intelligence of self-programming and decision making to adapt to changing surroundings without human supervision.

Exciting applications in which the presence of intelligent surfaces is fundamental are the creation of holograms and the creation of so-called invisibility cloaks, thanks to their ultrathin structure properties, programmability, and intelligence.

The growing need for wireless information transfer has stimulated research to identify solutions that can implement the efficiency of such networks. For example, metasurface-assisted wireless communications improve performance. Two solutions are possible: one in which radio signals, which are emitted by the transmitter and dispersed in space, can be retrieved and directed toward the desired user improving the SNR ratio, and the other in which additional information is encoded by the intelligent surfaces and transferred to users.

Three new architectures have been identified:

***Non-modulated metasurface backscatter communication (NMMBC)*** systems function as conventional wireless systems in which there is an RF vector to transport information and a mixer in which the modulation and demodulation of the signal are carried out. The presence of the intelligent metasurface can be considered an extension of antenna arrays in conventional systems; it serves, in practice, to increase the number of information channels.

***Modulated metasurface backscatter communication (MMBC)*** systems can be applied to overcome the problems related to the possible interception of information distributed in space without radiation directions, typical of the previous architecture. In these systems, the intelligent metasurface that carries the information directly modulates the radio signal coming from RF, simultaneously playing the role of mixer and antenna.

***Ambient modulated metasurface backscatter communication (AMMBC)*** systems have no need for dedicated RF sources or a new frequency spectrum because the emitter can be an environmental RF source of the type of TV tower, Bluetooth, cellular base stations, or Wi-Fi. The metasurface plays three roles: mixer, antenna, and energy collection collector. It has the advantage over traditional ambient backscatter systems, ensuring secure multiuser communications and higher transmission speeds.

Intelligent surfaces have also proven efficient for intelligent sensing by enabling the connection between the physical and digital worlds. Three techniques are possible:

- Nearly digital-computing-free intelligent sensing.

A reprogrammable deep imager represents a usable model in which the metasurface is trained with data that can be obtained from the principal components analysis (PCA). With this method, it is possible to obtain the radiation patterns as required by machine learning at the physical level. The intelligent metasurface, in this case, performs a physical calculation function by producing the PCA characteristics from the input of the raw data as in an analog calculation. Therefore, the detection is carried out almost without digital processing.

- Hybrid-computing-based intelligent sensing.

An approach can be adopted on the basis of applying an analog preprocessing phase of high-dimensional data with an intelligent surface at the physical level, followed by a digital postprocessing phase carried out with deep neural networks at the digital level. On the basis of this criterion, Li et al. [77] proposed using smart EM cameras by integrating ANNs into intelligent metasurfaces to detect the movements of the hands and breathing of the subjects. The intelligent EM camera detects people in real time in a scene with high spatiotemporal resolution, and checks the EM wave fields toward the points of interest to better recognize the points of the body.

- Hybrid-computing-based intelligent integrated sensing.

Data acquisition at the physical layer and postprocessing of data at the digital level are integrated as a whole and are learned simultaneously. Two measurement networks have been designed: a measurement network called m-ANN and a reconstruction network called r-ANN, jointly optimized to extract information in a variational autoencoder.

### 6.2. Related Work

Different experiences in which the combined study of HAR and IPS is addressed are reported. For example, Jamil et al. [72] developed a compensation mechanism, EPBCM, to improve location prediction by reducing drift and position errors caused by navigation algorithms. The model incorporates three algorithms: the HMM module for detecting accelerometric values for activity recognition and two others for localization based on PDR and BLE beacon. An unscented Kalman filter (UKF) is used in estimating position and orientation. Inertial sensors from a smartphone were used for data collection. The HMM model was applied to inertial data to recognize the activities carried out by the owner of the mobile device. Through a 3D navigation system, the movements of the subjects were traced. For the calculation of proximity between the smartphone and the fixed position of the beacon, the average weighted centroid localization algorithm (AWCLA) was applied. The EPBCM algorithm uses an estimation combiner to combine the position coordinates of the two position algorithms to obtain a position with less error. Localization based on the BLE beacon is applied as a compensation algorithm. The process is divided into several steps through which the data is filtered. On the sensory data, the orientation estimation is carried out with the help of the UKF.

The Kalman filter is applied to the beacon signals for noise cancellation, attenuation of RSS measurements, and integration of raw measurements of the signals. The authors developed several performance analyses to ascertain the significance of the proposed mechanisms, such as position accuracy, comparison between performed and estimated tasks, and validity of orientation estimation based on AHRS and UHF. The results of HMM-based activity detection were verified with the K-mean clustering technique. The comparison between the real data of six different locations and those estimated with EPCM revealed an error ranging from 0.01 to 0.65 depending on the reference position. A comparison was also made between the positions estimated with the three algorithms (EPBCM, PDR, and BLE beacon) and the real ones. XEPCM’s estimates were the most accurate. To evaluate the performance of the activity tracking system, the trajectories of the route taken from one floor to the upper floor using two flights of stairs were displayed. The analysis confirmed the effectiveness of the tool. Several pedestrian walking experiments were performed to further evaluate performance and compare the smartphone’s position with the various landmarks along the way. The estimated trajectory differed slightly from the real one.

Vandewiele et al. [78], as part of an innovative home project, introduced a system to recognize human activities using a network of sensors, including video cameras. The classic sensors of smart homes, dedicated to contact, temperature, motion, light, and any other device capable of connecting via a wireless communication medium, are distributed everywhere and can be used as constituent elements of behavior detection and recognition algorithms. The information provided by the cameras can be redundant but is helpful in case of malfunction of the sensors and is still useful in situations where several subjects perform actions simultaneously and the sensors cannot detect them on their own.

The application aims to recognize daily living activities in an elderly care context. The authors proposed a qualitative model for recognizing human activities on the basis of an unsupervised learning approach using episodic extraction techniques. Due to the variety of activities carried out in sequence, the authors focused their experiments on recognizing the “meal preparation” activity due to the system’s accuracy. The first results showed that the system could learn fragments of activities rather than entire activities. To measure the system’s performance, they extracted 13 episodes learned from the training data occurring when meals are prepared. Next, they tested the recognition system on the 11 sequences of accurate data, and 77% of these patterns were detected.

Moreira et al. [79] proposed an interesting application based on sensory data acquired through a smartphone and a convolutional long short-term memory (ConvLSTM) for the classification of human activities within an indoor environment network. They considered nine activities for classification. The authors associated the recognition system with a pre-existing positioning system with which they evaluated human activities considering different test paths in a multistory building obtaining an average positioning error of 2.4 m. Through inertial sensors, the internal positioning system can recognize the type of activity, e.g., a flat path or a climb of stairs. They also integrated the proposed HAR model with the fingerprint-based positioning system used by Guimaraes et al. [80]. A filter fusion system was also used to combine several sources of information. Moreover, more than 84% of floor transitions were correctly identified and performed in those routes, better estimating the user’s current position in a multistory building. The results showed that combining the recognition of activities with the positioning system improved the identification results.

Ruan [81], to create an innovative system in which individuals are not required to wear any device, proposed an approach for location and asset recognition using passive RFID tags. The project aims to help older people live longer independently and safely in their own homes, with minimal support from their caregivers. The author structured the application with three modules: the wireless RFID sensor network (WRSN), activity discovery (AD), and activity monitoring (AM). The WRSN module verifies the applicability of RFIDs and wireless sensors for identifying the location and activities of individuals. The sensory data and RFIDs collected with the previous module are extracted and grouped with the AD module to detect individuals’ routine activities automatically. Lastly, the AM module recognizes the actions and leases they are carried out. To achieve the intended goal, a series of passive tags and a reader equipped with antennas create an RSS field that ensures that the RSSI signal covers the area under consideration. RSSI vectors from known locations are used to train the classifier, which later predicts a position reported by a new RSSI vector. For the tracking phase, the author used a traditional kNN model enriched with probabilistic information to evaluate the probability of the positions obtainable from the RSSIs vectors used for constructing the emission matrix in the hidden Markov model (HMM). In the phase after obtaining the transmission matrix, the author applied the Viterbi search algorithm to determine the most likely path of the subject. For the reconnaissance phase of human activities, an array of passive RFID tags is placed within the area under consideration. Lastly, the basic principle of sparse representation is applied to create the activity recognition model. With the help of dictionary-based learning, structural information is identified among the RSSI signals of the different activities.

Meanwhile, a feature selection method was used to extract signal patterns based on filters for canonic correlation analysis. The last phase of the study focused on analyzing the activities related to the residential context, verifying whether the elderly usually carry out their daily activities or are found abnormal, such as requiring timely assistance. For example, an activity reasoning engine based on a suitable sensor based on Open Cyc, the world’s largest and most complete common-sense knowledge base with more than 300,000 concepts, has been developed for this phase. The study did not report the accuracy values found in the estimates made.

Furthermore, Dao et al. [82] adopted an integrated system based on UHF RFID passive tags and KNN method. A landmark reference tag grid was applied in their system. The RSSI tag is the principal value used to determine the location of the target tags using the K-nearest neighbor (KNN) algorithm. Their solution is based on a low-cost system that uses a small number of passive reference tags and a single antenna reader to locate objects and mounts a mobile tag. Information about the tag is stored in a file containing a number, ID, collection time, and RSSI value of collected tags. The path loss is applied to construct an error map for all reference locations based on this data. Subsequently, this error map and RSSI values are used in the context of the KNN method to locate the object’s coordinates. The reference tag grid based on the landmark model includes 28 reference positions. The target positions are distributed randomly in this tag grid. The RSSI value of tags is collected at a fixed antenna position. The process for the RSSI collection in the database is divided into two phases: a first phase in which the data of all the reference tags are collected, and the second phase which is dedicated to collecting the target position data. The measurements for each of the target positions as for the reference positions are repeated 10 times. The process is systematically repeated from the first to the last target position. The results obtained showed a localization error of about 32 cm.

Guo et al. [83] proposed an articulated platform based on the use of sensors on smartphones to identify the subjects’ activities, as well as to identify the internal environment. The project does not require additional devices. They combined pedestrian dead reckoning (PDR), human activity recognition (HAR), and landmarks to acquire accurate indoor location information. The authors applied a hidden Markov model to deduce the user’s initial position. From the detection of the activities of individuals, an indoor semantic landmark model was created to study the activities and trajectories. To estimate the subject’s position, the PDR is initially applied, to which landmarks are later added to correct the position. With the phone’s sensors, HAR is used to identify user activity. In addition, with HMM, the user’s initial position is then estimated. The PDR–smartphone system, with the help of inertial and orientation sensors, allows for tracing the user’s trajectory. The process includes step detection, step length estimation, direction estimation, and trajectory correction. As a supervised learning method to deduce user activities from sensory data, the KNN algorithm is applied, and, with the help of landmarks, the accuracy is then improved. As landmarks are used as the key points in a trajectory, a landmark list can denote a trajectory. There are three types of landmarks added to the list: stairs, turn, and doors. Adding semantic information to the landmark and the adjacent segment, we get a semantic description of a trajectory. Experiments conducted to fully evaluate the proposed approach not only showed high localization accuracy but also highlighted the effect of the landmark on location accuracy. The accuracy of the classification was greater than 99% in detecting stairs and walking activities. Lastly, the results on the localization error showed that the PDR errors increase with distance, and a high average localization accuracy (0.59) was achieved; they used the landmark to correct the cumulation errors.

Wang et al. [84] designed an original application with a model based on Wi-Fi fingerprints starting from the consideration that people’s behaviors can influence the propagation of the Wi-Fi signal and introduce specific patterns in Wi-Fi signals, called Wi-Fi fingerprints, which can be further explored to identify human activities and locations. They proposed a novel deep learning framework for joint activity recognition and indoor localization using Wi-Fi channel state information (CSI) fingerprints. Although there are numerous applications in which the Wi-Fi CSI has been studied for human activity detection, there is a lack of evidence that the joint study of activity recognition and internal localization has been addressed. They proposed a novel one-dimensional convolutional neural network (C1D) based on ResNet [85], comprising two branches, one for recognizing activities and the other for internal localization. To verify the validity of the model, they used the IEEE 802.11n protocol with two USRPs (universal software radio peripherals) and with a dataset appropriately prepared for a potential human–computer interaction application. The dataset contained six hand gestures: hand up, hand down, left hand, right hand, hand circle, and hand cross. The test was carried out by a volunteer who repeated these activities 15 times in each 16 different locations, realizing 1394 samples (after excluding the invalid data). This produced different Wi-Fi fingerprints when performing the same activity but at different locations and activities in one location. The system contains two sets of personal computers and USRPs, which work as Wi-Fi transmitters and receivers. Lastly, an Ettus clock synchronizes the two sets. The experimental results confirmed the model’s validity by reporting accuracy values of 88.13% on the recognition of the activity and 95.68% on indoor localization.

Fiorini et al. [86] proposed a well-organized work in which they designed a system with body information, vital signs, and the user’s internal position aggregated to improve the recognition of activities. The proposed model integrates wearable sensors capable of monitoring cardiac activity (electrocardiogram, ECG), body posture, and acceleration of the lumbar area and an environmental localization network capable of estimating the user’s position. The localization network is implemented in such a way as to locate both range-free and range based. From an architectural point of view, the system consists of a hardware layer, a communication layer, and a data processing module. The hardware component consists of three sensors: an STMicroelectronics iNEMO-M1 inertial sensor, a Zephyr Bioharness (BH3) Bluetooth chest strap that can monitor the ECG, and ZigBee wireless location sensors. The network configuration consists of a ZigBee coordinator (ZC), a data logger (DL), a wearable mobile node (MN) equipped with an omnidirectional antenna, and a set of ZigBee anchors (ZAs). The ZAs are installed in a fixed position in the experimental site on the walls and inside the furniture to detect the location within the rooms accurately and is equipped with 60° sectoral antennas to improve the SNR ratio of RSS signals. Each ZA calculates the user’s position as a function of the RSS signal exchanged between the nodes and transmitted this value to the DL. The communication is based on the ZigBee module for localization and the Bluetooth module for ECG.

As far as the processing system is concerned, a PC collects all the data through four modules. One module for capturing ECG data, one to collect data from inertial sensors, two to collect RSS data from DL, and another to calculate the user’s position. Three supervised machine learning algorithms were used for the classification: decision tree (DT), support vector machine (SVM), and artificial neural network (ANN). Two classification models were created for each algorithm, one containing information about the user’s location to assess whether the user could improve accuracy and the other without location information. Two techniques were used to validate the models: fivefold cross-validation (5CFV) and LOSO. The training dataset was created using nine subjects, while the test dataset included an unknown subject to testing how good the model is in the presence of new data. The model was ultimately tested in a realistic environment with a total of 3279 samples taken from 10 users to recognize the following activities: sitting to work at the PC, sitting to watch TV, lying on the couch, sitting in the kitchen, and sitting in the bathroom. The results showed that the three models, while presenting different accuracy values, all showed clear improvements with the information on the location. The DT went from 0.924 to 0.999, the SVM went from 0.995 to 0.999, and the NN went from 0.839 to 0.917.

Redondi et al. [87] developed an interesting system based on wireless sensor networks (WSNs) to supervise patients within a nursing home. It provides two main features:

- Monitoring of the patient’s status. Various information is collected on the patient’s status concerning standing, walking, supine, and prone activities.
- Localization and tracking of patients. The exact knowledge of the patients allows a quick intervention of the assistants in case of need.

The architecture of the system consists of the following elements: a personal localization and personal system (PLTS) module, a personal monitor system (PMS), and a network architecture (NA) composed of fixed nodes (AN) and mobile devices (MN) mounted on patients to provide information to be sent to an automated central controller. The PLTS module uses a localization algorithm based on the intensity of the received signal (RSS). The PMS uses a decision tree-based classifier (DT) to recognize acquired movements correctly. The PLTS module, to improve localization and tracking, uses particle filtering to “smooth down” the fluctuation of the RSSI sample. The personal monitoring algorithm is based on hierarchical DT, where the classification output is perfected at each tree level; general classifications are carried out in the highest levels with low-effort threshold decisions.

In contrast, more detailed classifications are carried out in the lower levels. The algorithm uses signals from a biaxial accelerometer linked to the waist to classify the movement. A window-by-window classification scheme has been adopted in which the movement is classified by analyzing the data collected in a 1 s window. Once the two components of the acceleration have been separated, the acceleration signal is examined to see if the patient is in a state of movement or rest. Since the energy spent during movement is greater than the rest activity, the tests are carried out for a fixed threshold value that the authors have placed equal to 0.2 in their tests. The authors used the acceleration value to detect a possible fall, starting from the consideration that if the acceleration takes a value more significant than the normal one equal to 1 g, this value can indicate a critical event such as fall detection. Experimental values showed that the accuracy of the localization system was about 2–3 m with a node arrangement still approximately equal to 0.15 knots/m^2^. As for the accuracy of the movements, the values were almost all close to 100.

Bibbò et al. [88] designed an innovative home care system for the elderly and pathological conditions. The system provides services to assist an integrated system for older adults. They developed the system architecture on an IoT platform capable of guaranteeing three functions:

- Data collection connected to the patient activity such as inertial, physiological, environmental, and localization data;
- Design of a convolutional neural network for activity recognition;
- Identification of the exact elderly position.

In order to verify the classification level of the AI model, the authors applied virtual reality technology [89]. The system’s core was the STM32L475 microcontroller IoT node, which embeds a network of sensors such as an accelerometer, gyroscope, magnetometer, temperature sensor, and proximity sensor, which belongs to the MCU based on the core Arm Cortex-M4. It also integrates wireless connectivity such as Wi-Fi, NFC, BLE, and sub-GHz bands. The Kalman filter algorithm then fuses the data collected by the sensors; the data are sent to the CNN for classification. The authors used the Keras model to design the CNN architecture trained on the wireless sensor data mining (WISDM) dataset. Thirty-six people collected the data through the smartphone with a sampling of 20 values for every person. The activity recognized were upstairs, downstairs, sitting, standing, and walking.

The neural network was composed of two convolutional layers, two ReLu layers, two pooling layers, and two fully connected layers. In order to detect the exact position of the people, the system was integrated with an ultrasound network made by beacons and mobile nodes. The beacons are placed at the corners of a square 4 × 4 × 3 m^3^ at the top of the ceiling while the patient wears the tag. The localization system is based on an RF synchronization between transmitter and receiver, an ultrasonic chirp signal for measurement of the distance, and the multilateration process to compute the distances. Distances are calculated by measuring the time of flight, the time elapsed from the transmitter to the receiver. The communication system uses the ANT protocol (wireless communication protocol of ANT wireless), which is not affected by interference in the transmission between different devices. The authors created a script to configure and establish the communication on the same Wi-Fi network between the server on which the patient’s coordinates are recorded and the system board that represents the client to which the information on the exact position of the subject is to be transferred. The results showed that the accuracy of the recognition of the assets was greater than 99%. In contrast, the accuracy of the positioning was of the order of cm in consideration of the accuracy of the measurement that was equal to about 1.7 mm, which we can consider an accurate value as it is greater than the limit value obtainable with the Cramér–Rao formula (CRB) [90]. Table 5 shows a comparison of the systems analyzed.

**Figure 16:**
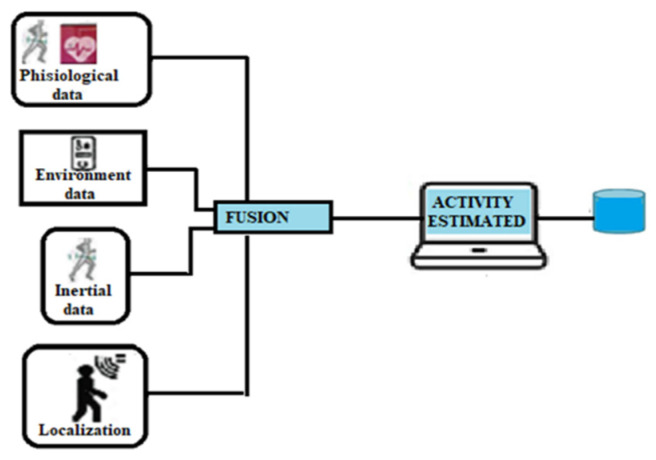
HAR/IPS system.

From the analysis of the solutions, we can observe no standard solution was adopted by the designers. Combining IPS with HAR improved prediction accuracy. The mainly applied technology was Wi-Fi with the use of inertial sensors. In addition, for the prediction of activities, the use of ML models that allow obtaining high values of accuracy was preferred. Figure 16 shows an integrated HAR and IPS system.

## 7. Discussion

The approach to this work was that of qualitative research, starting from the analysis of the state of the art of technologies, techniques, and algorithms that have carefully sought indoor positioning systems that can be applied in the tasks of localization and assistance to older adults. The completeness of the analysis was also examined from the point of view of the main evaluation metrics. Considering the requirements for home care applications that require good levels of accuracy, short return times, and less computation, it was observed that there are not many testimonies on this issue in the literature. We believe that combining location information and tracking, inertial, physiological, and environmental data can assist in creating an efficient home care system. Together with intelligent systems that can identify the activities carried out, the use of aggregate information on the positions and movements made by the elderly allows accurate monitoring of people in need of assistance. Through the combined use of this information, caregivers can assess abnormalities and deviations from habitual behaviors that may indicate a decline in the elder’s abilities. The exact knowledge of the position also facilitates interventions in emergencies and avoids potentially dangerous situations. The growth in the number of elderly people in need of care who wish to continue living in their homes while maintaining their habits has stimulated the creation of increasingly innovative home care systems to avoid hospitalizations with apparent savings in care costs. New technologies with significant advances in accuracy and speed, as well as the availability of smartphones equipped with integrated technologies such as Wi-Fi, Bluetooth, IMU, and high-definition cameras, can be used to improve the quality of location systems. However, integrated systems for elderly care require performance that is incompatible with long processing times and solutions that use special devices and specialized infrastructures. Acquisition, installation, and maintenance costs must be low in order for them to be accepted by users. No solution can satisfy the use of an IPS system in all indoor scenarios. Therefore, the problem of indoor positioning requires further research to obtain cost-effective solutions with good performance. IoT technology and hybrid architectures open up a broad scenario in which designers can experiment with new approaches to fill the existing gaps.

With the advent of IoT, recent technologies can provide various solutions to collect and transmit data, including location information. LTE and 5G broadband cellular networks are, in fact, suitable technologies for IoT environments. However, it must be considered in the design that the presence of heterogeneous devices significantly affects the system’s performance.

Innovative applications that integrate inertial sensors with wireless technologies can represent an exciting approach for scalability, accuracy, and cost. It is a solution that has the advantage of locating the position of all Wi-Fi devices without the installation of additional hardware and software, and there is no need for the line of sight.

The combination of different technologies represents a valid solution to improve system accuracy. It allows using the best features of each to find the most suitable solution to satisfy the adaptability in different indoor scenarios, heterogeneity of the network components, energy consumption, and accuracy. However, the use of multiple technologies can require more memory and computational time, affecting the cost.

In general, a localization network can use additional information from one or more sensors, additional data obtained from radio systems, or additional position estimates obtained from nearby references. An aspect to consider is the multipath effects and the reflection signals present during the transmission of the signal in closed environments.

The guiding principle should be to use data already available so as not to increase nodes’ costs or resource requirements in a pre-existing network. The two most critical decisive factors in the choice of the localization system are accuracy and price. However, the question of the impact of the localization scheme on network performance should not be overlooked. For example, the choice of passive beacons or active transmitters could, at best, slightly interfere with communication or, in the worst case, impose a severe limitation on the network’s communication capabilities.

## 8. Conclusions

The main contribution of this article was to investigate how different localization technologies have contributed to improving the accuracy in recognizing the activities carried out by the elderly within their home or in the nursing home. Location information is crucial to know the context in which the user is located to provide assistance services to improve the quality of life. In the literature, there are not many testimonials in which information on the patient’s position inside closed environments was combined with systems for recognizing daily activities. We presented a detailed description of the different indoor location detection techniques and commonly used technologies in the proposed revision. We also provided an analysis of the various solutions adopted, highlighting the distinctive aspects of each. In addition, each author chose their technological architecture on the basis of needs to be met and the parameters to be privileged (energy efficiency, precision, scalability, coverage, cost, and availability of resources). From the diversity of the solutions presented, it is clear that there is no standard solution, but each has its limits due to accuracy, sophisticated models, limited coverage areas, or high costs. The choice of the right solution for the hypothesized application depends on factors influencing the system’s performance. This study can provide an evaluation scheme for the choice of the suitable solution for realizing an integrated application for home care for the elderly. The solution to be adopted must derive from a balanced compromise among the following parameters: cost, available resources, characteristics of environments, required accuracy, and computational complexity.

The advent of IoT technology and connectivity improvement will allow hybrid solutions in which it will be possible to combine different technologies to improve system performance.

## Figures and Tables

**Table 1:** Range of accuracy (meters) and coverage (meters) for technology.

Parameter	Wi-Fi	Ultrasound	Infrared	Bluetooth	Rigid	ZigBee	UWB
Accuracy	1–10	0.01–0.1	5–10	2–15	0.5 (passive) 1 (active)	1–5	0.1–1
Coverage	20–50	2–10	1–5	1–30	1–100	10–100	0.50–10

**Table 2:** Comparison of signal measurement techniques.

Technique	Accuracy	Cost	Advantages	Disadvantages
TOA	High	High	Scalability, does not require any fingerprint	Needs time synchronization, difficult to implement, produces multipath effects
TOP	High	High	No need time synchronization among devices and received nodes, does not require any fingerprint	Requires time synchronization between the received nodes, difficult to implement in narrow bandwidth, multipath effects
RTT	High	High	Does not require time synchronization, low complexity	Affected by multipath effects and noise, different processing time delays
AOA	Medium	High	No need for any fingerprint, no need for time synchronization, low number of APs	Requires additional directional antennas, decreases in accuracy as distance from source increases
RSS	Low	Medium	No need for synchronization, can be used with different technologies, easy to implement	Suffers from multipath effect, noise, can require fingerprint

**Table 3:** Comparison of indoor localization systems.

Technology	Measure Method	Cost	Advantages	Disadvantages	Accuracy (m)
RFID	Proximity, RSS TOA, TDOA, AOA	High	Does not require LOS between TR and RT, simultaneous and fast reading of multiple tags	Small coverage, multipath effect and signal fluctuation, limited capabilities of passive tags	0.5 (passive) 1 (active)
WLAN	RSS, TDOA	Medium	Does not require LOS, presence in multiple buildings, medium scalability	Complex methodology, system redesign in case of changes in the environment	10–50
Bluetooth	TDOA, RSS	Low–medium	Good accuracy, no need additional infrastructure, does not require LOS, present in most smartphones	RF interference, limited coverage and mobility	2–15
UWB	TDOA	High	Low energy consumption, high accuracy, passes through walls and any other obstacles	Needs time synchronization, limited coverage, performance degrades in NLOS.	0.1–1
ZigBee	RSS, AP ID	Low	Low power consumption	Requires special equipment, vulnerable to interference caused by a wide range of signal types	1–5
Ultrasound	TOA, TDOA	Medium	Good accuracy, not affected by multipath	Interference by high-frequency sound, loss of signal for obstruction	0.01–0.1
Infrared	AOA, TOA, TDOA	Medium	Low power, no multipath effect, medium accuracy	Does not penetrate walls, requires LOS, sunlight interference, short range	5–10
Magnetic	AOA, TOA	Medium	Medium power consumption	Requires magnetic field mapping, errors increase with the size of the fingerprinting map	1–3
Optical	Scene analysis, proximity	Medium	Performance improvement by fusion of image data with data from other sensors	The transformation from the image space into the object space requires additional depth information	0.1
Inertial	Dead reckoning	Low	Great reliability, reduced size	Cumulative errors, high complexity	Error range 0.5–2% total traveled distance

**Table 4:** Comparison of HAR technologies.

Typology	Advantages	Disadvantages	Accuracy
Visual-sensor	Ease of use, ease of analysis from images, data reliability, alternative to multiple sensory devices,	Privacy, sensitive to environmental conditions, higher cost, increased processing power, longer processing time	99%
Non-visual sensors	Detection of any information about behavior, no privacy issues, lower cost, less processing power, lower power consumption, less processing time	Need for a large set of sensors, data reliability, system vulnerability due to sensor malfunction, lower accuracy values	70–80%
Multimodal sensor	Suitable for the collection of data of different nature, lightweight devices, lower power consumption, less processing time	Need for multiple sensors, acceptance issues, need to wear sensors, efficient fusion algorithms	99%

**Table 5:** Comparison of HAR-IPS systems.

Author	Adopted System	Technology	Technique	Accuracy
Jamil [72]	Inertial sensors of smartphone	PDR-BLE	EPBCM/HMM	99%
Vandewiele [78]	Cameras and smart home sensors	Visual/Wi-Fi	Unsupervised model	77%
Moreira [79]	Inertial sensors of smartphone	Fingerprints	ConVLSTM	84%
Ruan [81]	RFID	Wi-Fi	KNN	Not declared
Dao [82]	UHF/RFID Landmark	Wi-Fi	KNN	32 cm error
Guo [83]	Inertial sensors of smartphone	PDR	KNN	99%
Wang [84]	Inertial sensor	Wi-Fi fingerprints	C1D	88% recognition, 95% localization
Fiorini [86]	Inertial–physiological sensor (ECG)	Bluetooth	DT/SVM/ANN	0.924–0.994 DT 0.995–0.999 SVM 0.839–0.917 ANM
Redondi [87]	Anchors/mobile device	Wi-Fi	DT	99%
Bibbò [88]	MEMS/ultrasound	Wi-Fi	CNN	99% recognition 1 cm localization

## Data Availability

Not applicable.
